# Effect of COVID-19 on ^18^F-FDG PET/CT: Is There a Need to Consider COVID-19 Status Before Planning ^18^F-FDG PET/CT for Oncologic Evaluation?

**DOI:** 10.2967/jnmt.121.262145

**Published:** 2021-09

**Authors:** Anwin Joseph Kavanal, Santosh Ranjan Jena, Rajender Kumar, Chandan Krushna Das, Sunil Kumar, Bhagwant Rai Mittal

**Affiliations:** 1 Department of Nuclear Medicine, Postgraduate Institute of Medical Education and Research, Chandigarh, India; and; 2 Medical Oncology OPD, Department of Radiotherapy, Postgraduate Institute of Medical Education and Research, Chandigarh, India

**Keywords:** COVID-19, ^18^F-FDG PET/CT, lung metastasis, response evaluation, renal cell carcinoma

## Abstract

Incidental detection of coronavirus disease 2019 (COVID-19)–related lung changes on ^18^F-FDG PET/CT images of oncology patients has been increasingly reported. Most of the case reports or series have stressed the retrospective diagnosis of COVID-19 with the help of ^18^F-FDG PET/CT lung findings. In this case report, we introduce a different aspect of COVID-19–related lung changes on ^18^F-FDG PET/CT, interfering with the evaluation of metastatic lung lesions in a patient with renal cell carcinoma.

Various lung involvement patterns have been reported on ^18^F-FDG PET/CT scans of coronavirus disease 2019 (COVID-19) patients undergoing workup for various malignancies. The patterns range from ^18^F-FDG–avid diffuse ground-glass opacities to ^18^F-FDG–avid patchy consolidatory changes, with or without ^18^F-FDG–avid mediastinal lymph nodes, depending on the imaging time from the onset of infection and other unknown factors (*1*–*4*). COVID-19 infection was a retrospective diagnosis in most reported cases, after the typical findings were seen on the ^18^F-FDG PET/CT images (*3*–*7*). Here, we present a different aspect of COVID-19 on ^18^F-FDG PET/CT, in which there was interference with response assessment in a patient receiving chemotherapy for pulmonary metastasis from renal cell carcinoma.

## CASE REPORT

**FIGURE 1.**
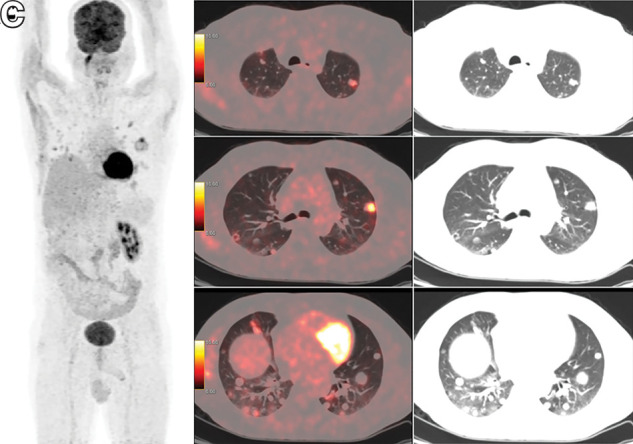
^18^F-FDG PET/CT whole-body maximum-intensity-projection image (A), axial PET/CT images (B), and corresponding CT images (C) showing variably ^18^F-FDG–avid random nodules in both lung fields (SUV_max_ of hottest nodule, 9.9).

**FIGURE 2.**
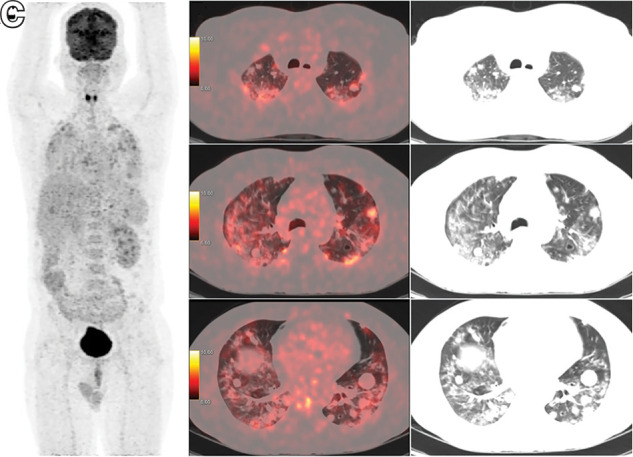
^18^F-FDG PET/CT whole-body maximum-intensity-projection image (A), axial PET/CT images (B), and corresponding CT images (C) showing ^18^F-FDG–avid diffuse ground-glass opacities/patchy consolidatory changes bilaterally in lung fields from apex to base, obscuring details of metastatic lesions (SUV_max_ of hottest nodule, 7.8; SUV_max_ of ground-glass opacities, 7.3).

A 45 y-old man with a known case of metastatic renal cell carcinoma underwent cytoreduction nephrectomy followed by first-line chemotherapy with pembrolizumab and axitinib because of multiple cannonball metastases in the lungs. ^18^F-FDG PET/CT at the end of treatment showed disease progression in the form of an increase in the number and size of lung nodules. The patient was then started on second-line chemotherapy with oral lenvatinib (18 mg daily) and everolimus (5 mg daily). His interim ^18^F-FDG PET/CT scan (Fig. 1) showed a favorable response (>30% reduction in size and ^18^F-FDG avidity compared with baseline PET/CT) to second-line therapy, and he was continued on the same treatment. He was diagnosed with COVID-19 in May 2020 on evaluation for malaise and chills. He was managed conservatively with antibiotics, antipyretics, and multivitamins in a local hospital. He had no symptoms or signs suggestive of pneumonia and never required oxygen support during the 11-d course in the hospital. He was discharged from the hospital after a negative nucleic acid test 1 wk before he was scheduled for an ^18^F-FDG PET/CT scan at 6 mo of chemotherapy to determine the response. The ^18^F-FDG PET/CT scan (Fig. 2) showed ^18^F-FDG–avid diffuse ground-glass opacities/patchy consolidatory changes bilaterally in the lung fields from apex to base, obscuring the metastatic lesions. The COVID-19–related lung changes obscured both the anatomic and the metabolic features of the metastatic lesions, leading to difficulty in assessing the response to treatment.

## DISCUSSION

^18^F-FDG uptake in ground-glass opacities in the background may add spill-in counts to metastatic lesions, causing a falsely high uptake in metastatic lesions (*8*). For this reason, an accurate assessment of the metabolic response was not possible in this patient. The patient was advised to repeat the nucleic acid test because of ^18^F-FDG avidity in the ground-glass opacities/consolidatory changes and was found to be positive. The patient was then advised to remain home in isolation again.

## CONCLUSION

During the COVID-19 pandemic phase, we have to consider sources of possible interference such as described in this report before scheduling patients for ^18^F-FDG PET/CT scans for various oncologic purposes.

## DISCLOSURE

No potential conflict of interest relevant to this article was reported.
